# A design of experiments study of nasal spray deposition in the alberta idealized nasal inlet

**DOI:** 10.1007/s13346-026-02147-0

**Published:** 2026-05-06

**Authors:** Mathilde Sophie Felding, Charlotte Cubbin, Claus Moser, Fatemeh Ajalloueian, Line Hagner Nielsen

**Affiliations:** 1 Department of Health Technology, Technical University of Denmark, Ørsteds Plads 345C, Kgs. Lyngby, 2800 Denmark; 2 Department of Immunology and Microbiology, Costerton Biofilm Center, University of Copenhagen, Blegdamsvej 3B, Copenhagen, Denmark; 3 Department for Clinical Microbiology, Rigshospitalet, Copenhagen University Hospital, Blegdamsvej 9, Copenhagen, 2100 Denmark

**Keywords:** Intranasal administration, in vitro nasal spray testing, nasal geometries, nasal cast, intranasal formulations, nasal deposition profile

## Abstract

Intranasal administration of drugs possesses numerous advantages such as easy and direct application, rapid onset of action and reduced risk of systemic adverse effects. The nasal cavity consists of several regions and especially the respiratory region, known as the turbinates, is considered favorable for drug uptake. Current Food and Drug Administration (FDA) guidelines for in vitro testing of nasal spray formulations recommend characterization of emitted dose, plume geometry, spray pattern, and particle size distribution. However, recommendations or guidelines for assessing regional deposition within the nasal cavity remain lacking. In this study, we used the Alberta Idealized Nasal Inlet for in vitro testing and a design of experiments (DoE) approach to test the regional deposition of a commercially available nasal spray Otrivin^®^. We show that three main parameters (airflow, angle of administration and temperature) play vital roles in the deposition pattern. Furthermore, we uncovered significant effects of administration angle and temperature, as well as combined interactions between airflow and angle, and temperature and airflow. Our results demonstrate the need for introducing a physiologically relevant temperature when conducting in vitro studies, and that combined interactions are vital to investigate and understand for assessing regional deposition of nasal spray formulations in vitro.

**Graphical abstract:**

**Figure:**
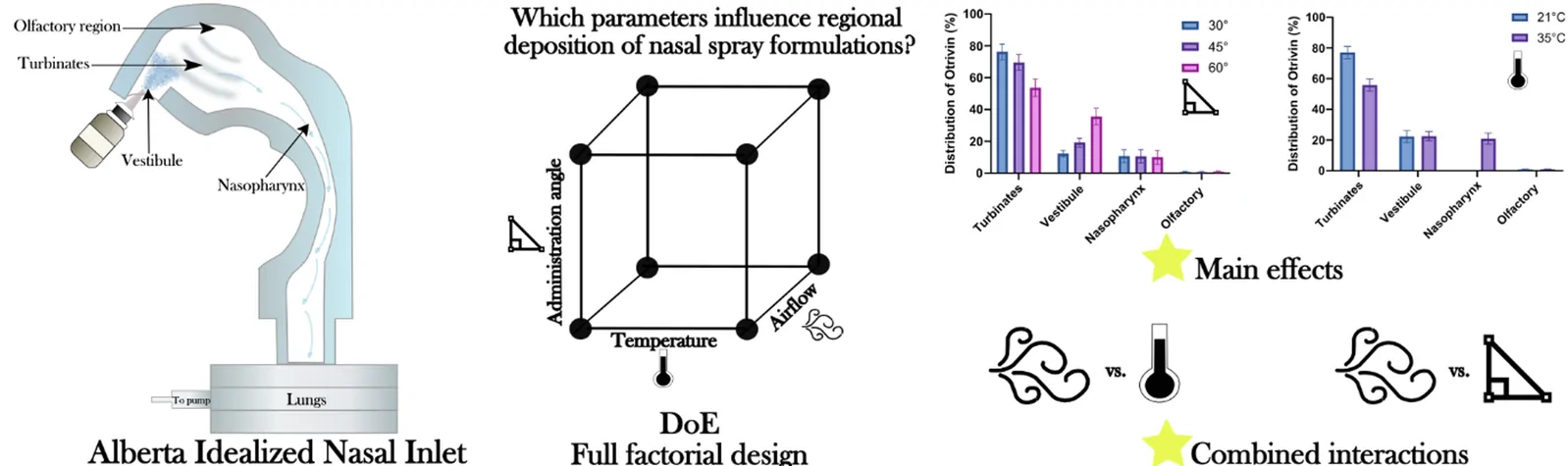

**Supplementary Information:**

The online version contains supplementary material available at https://doi.org/10.1007/s13346-026-02147-0.

## Introduction

Intranasal administration of drugs is a non-invasive delivery route with several advantages. These include ease of application, avoidance of first-pass metabolism, rapid onset of action, reduced risk of systemic adverse effects, and the possibility for both local and systemic delivery [1, 2]. However, to understand how these advantages can be exploited, it is important to consider the anatomy of the nasal cavity.

The nasal cavity consists of the nostrils, vestibule, atrium, respiratory region (turbinates), olfactory region, and nasopharynx. The respiratory mucosa is the most favorable site for drug uptake as it contains the superior, middle, and inferior turbinates situated along the lateral wall of the nose [1]. The turbinates warm and humidify inspired air. Its large and highly vascularized surface area (~ 120–150 cm^2^) makes it particularly suitable for drug uptake [3, 4].

Given these structural features, the design and characterization of nasal spray formulations are crucial for optimizing intranasal drug delivery. For a nasal spray formulation to have an optimal effect, it is vital that the formulation reaches the desired site. In the early stages of development, in vitro testing is especially valuable, as it enables fine-tuning of spray characteristics while providing essential feedback prior to in vivo evaluation [5]. Current guidelines from the Food and Drug Administration (FDA), for in vitro testing, recommend characterization of emitted dose, plume geometry, spray pattern, and particle size distribution [6, 7]. However, there is still a lack of proper recommendations or guidelines for assessing regional deposition within the nasal cavity [8].

Previous studies developed 3D printed nasal casts based on computed tomography (CT) scans of several adults to test in vitro regional deposition of nasal spray formulations [9, 10]. Additionally, other studies used transparent in vitro nasal casts coated with Sar-Gel. This changes color upon contact with water-based solutions and thereby, enabling capture of the distribution pattern using a digital camera [11, 12].

While various nasal cast models offer valuable insights into intranasal drug administration, a simplified yet anatomically representative, the Alberta Idealized Nasal Inlet (AINI; Copley Scientific, UK), has been developed for testing regional deposition of nasal spray formulations. The AINI is an idealized model of the nasal cavity designed to enable standardized in vitro testing [6]. The AINI can be separated into four regions, the vestibule, turbinates, olfactory, and nasopharynx. This segmentation allows for sample collection from each region and determination of regional nasal spray deposition. Previous studies have demonstrated that the AINI can reproduce average regional deposition patterns observed from in vitro realistic adult nasal geometries and in vivo scintigraphy studies [13, 14].

In this study, we used a design of experiments (DoE) approach to investigate how selected parameters influence nasal spray deposition of a commercially available nasal spray, Otrivin^®^, within the AINI. Specifically, we examined ^(1)^ spray cone angle, ^(2)^ inhalation flow rate, and ^(3)^ temperature, aiming to identify not only main effects, but also potential interactions between these factors. Different inhalation flow rates were chosen. 0 L/min was chosen to simulate breath holding, 7.5 L/min was to simulate slow breathing [13], 15 L/min was selected to simulate moderate breathing, whereas 29 L/min was to simulate heavy breathing (as recommended by manufacturer). Furthermore, we investigated the effect of using an internal Brij coating in the AINI to simulate in vivo conditions of the nasal mucosa and reduce particle bouncing [15]. We hypothesized that several parameters influence the regional deposition in the AINI and that these parameters could interact with each other. To our knowledge, this is the first study exploring how physiologically relevant temperature conditions and parameter interactions affect regional deposition in the AINI.

## Materials and methods

### Materials

Brij^®^ 35, glycerol and xylomethazoline HCl were purchased from Sigma-Aldrich (St. Louis, MO, USA). Phosphate buffered saline (PBS) tablets (Gibco™) were obtained from Thermo Fisher (Paisley, UK). Ethanol absolute was acquired from VWR International (Radnor, PA, USA). Otrivin^®^ (1 mg/mL xylomethazoline) (Haleon Denmark ApS, DK) was bought from a local pharmacy. Purified water (MilliQ, Millipore) (MQ) was used throughout the study.

### Preparation of Brij coating

Brij coating was prepared according to the protocol described by [16]. Brij^®^ 35 was dissolved in ethanol to achieve a final concentration of 15% (w/v) and stirred until fully dissolved. This solution was added to glycerol in a 1:5 ratio and stirred at 37 °C. The final solution was stored at 4 °C.

### Design of experiment setup

A full factorial design with duplicates was created and the order of the tests was randomized. Using Otrivin^®^, and a full factorial design, we tested spray cone angles of 30°, 45°, and 60°; inhalation flow rates of 0, 7.5, 15, and 29 L/min; at two temperatures (21 °C and 35 °C). Finally, we tested the effect of applying a mucosa-simulating coating in the AINI at 30°, 29 L/min, at both 21 °C and 35 °C. The experiments performed are summarized in Table 1.

**Table 1:**
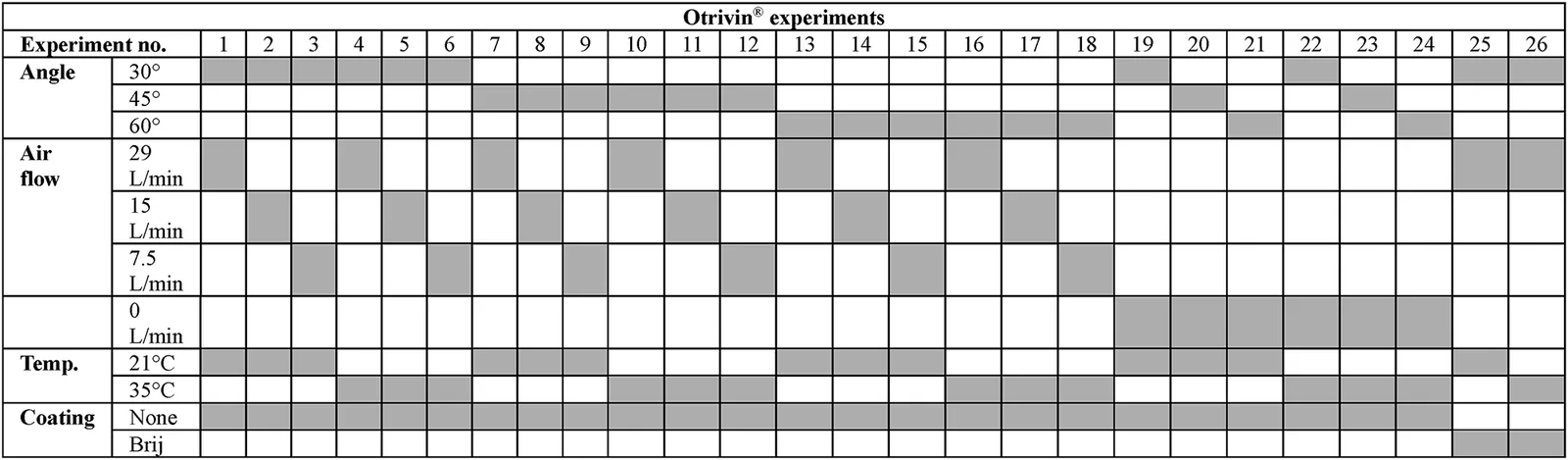
Overview of the experiments conducted in this study. The grey boxes indicate the parameters for each experiment. Each experiment was performed in duplicates and randomized

### Setup of the AINI

**Fig. 1:**
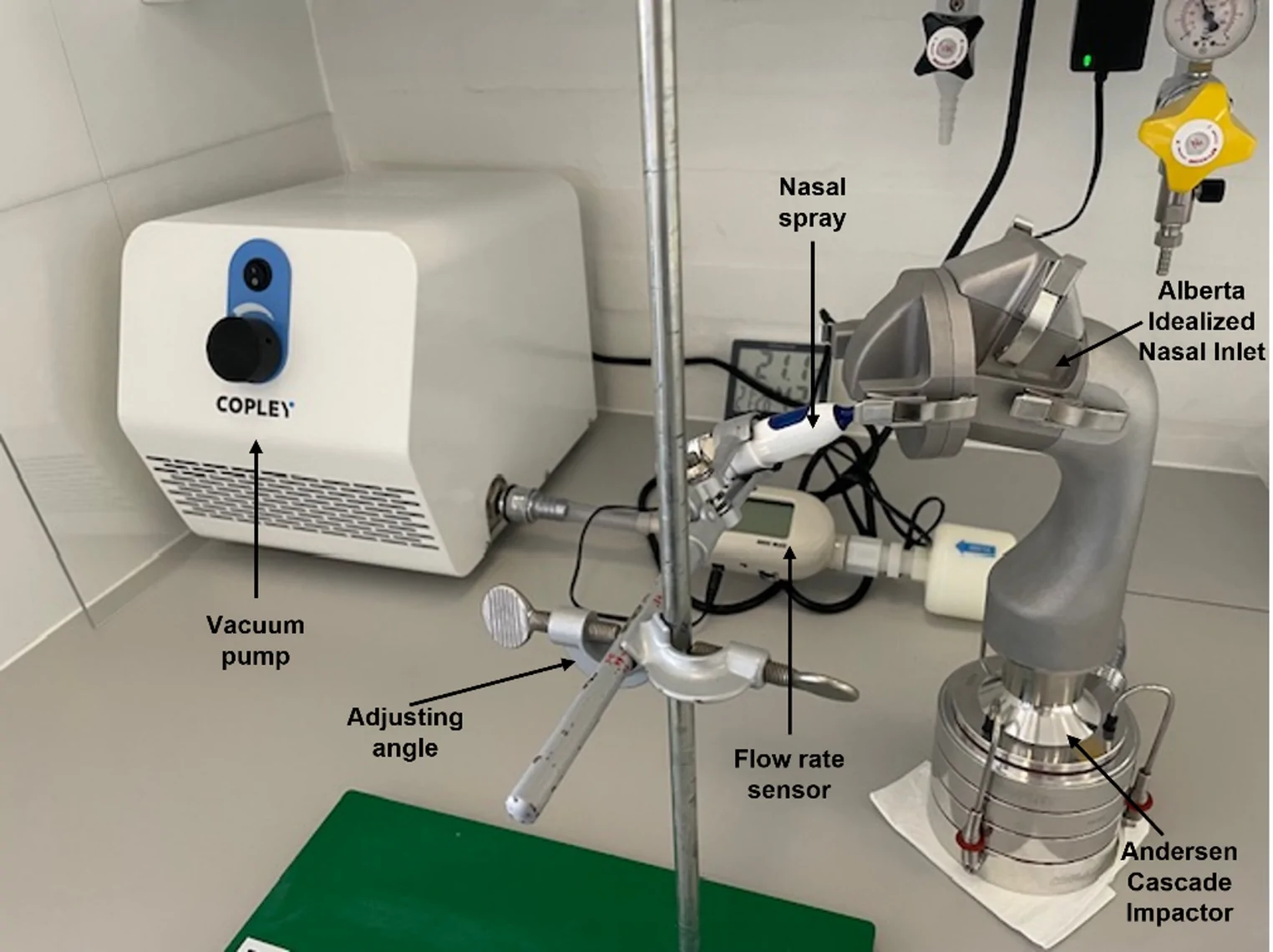
Experimental setup. The vacuum pump was connected to the flow rate sensor, which was connected to the Andersen Cascade Impactor and the AINI. The nasal spray was positioned at the in-house designed holder which could be adjusted to the correct angle from the horizontal plane

Regional nasal deposition was evaluated using the AINI model (Copley Scientific, UK) coupled with the Andersen Cascade Impactor (ACI) (Copley Scientific, UK). The AINI-ACI assembly was connected to a flow rate sensor (FRS) (Copley Scientific, UK), which was in turn connected to a vacuum pump (Copley Scientific, UK). A custom-made nasal spray holder and angle adjuster were designed in-house, and the angle was measured from the horizontal plane (Fig. 1).

For experiments performed at 35 °C, the AINI was preheated in an oven at 70 °C for 5 min. A temperature of 35 °C was chosen due to the slightly lower temperature observed in the nasal cavity compared to the rest of the body [17]. The temperature was monitored with an infrared thermometer until the target temperature (35 °C) was reached. Temperature readings were recorded from the nostril before and after each experiment. For experiments conducted at room temperature (RT), the ambient temperature was recorded at the exact time of testing.

For experiments with Brij coating, the AINI was prepared by immersing a pipe cleaner in the Brij solution, and this was distributed along the internal surfaces of the regions, to create a coating layer distributing along the surfaces of the nasal cast. Coating experiments were performed at 21 °C and 35 °C with a 30° angle of administration and 29 L/min airflow.

Prior to nasal spray administration, the vacuum pump was allowed to warm up for a few minutes. The pump was then adjusted to the desired airflow and recorded for each experiment. For experiments at 35 °C, during the vacuum pump warm-up and airflow calibration phases, a custom-designed thermo-hood was used to cover the AINI and ACI to minimize thermal loss and maintain a stable internal temperature. The nasal spray was positioned at a 30°, 45° or 60° angle from the horizontal plane, and an airflow of 0, 7.5, 15–29 L/min was applied.

### **Actuation of Otrivin**^®^

Following the setup of the AINI, nasal spray and adjustment of airflow, the nasal spray was actuated into the AINI through the nostril opening. The vacuum pump was kept on for 10 s following the actuation, after which it was turned off. For experiments at an airflow of 0 L/min, the vacuum pump was turned off during the actuation and for 10 s afterward, then subsequently turned on for 10 s.

### Sample collection

Regional openings of the AINI were sealed as outlined in Table 2, depending on the experiment type. Sample collection from each region was done according to the following procedure. A representative illustration of the regional openings and sample collection can be seen in Figure S1.

**Table 2:** Overview of the sealing types used for sample collection, depending on the type of experiment

	Sealing type
Experiments at 35 °C	Tape
Experiments with coating	Duct tape & parafilm
All other experiments	Parafilm & tape

Vestibular region: the interface facing the turbinate region was sealed with tape, 2 mL of MQ water was introduced through the opposite opening, and the inlet was sealed with parafilm.

Turbinate region: connections to the olfactory and nasopharyngeal compartments were blocked with tape, 2 mL of MQ water was added through the remaining opening and sealed with parafilm.

Olfactory region: two-thirds of the opening was sealed with tape, 0.4 mL of MQ water was added, and the remaining portion was sealed.

Nasopharyngeal region: the interface to the ACI was sealed with parafilm, 2 mL of MQ water was introduced through the opposite opening, and the inlet was sealed.

Nasal spray recovery from each region was achieved by manual agitation to promote uniform sample dispersion while preventing leakage. After agitation, the seals were removed, and the diluted sample was collected into a Petri dish. Any visible droplets were recovered and combined with the bulk sample.

After each experiment, the AINI was rinsed with ethanol to eliminate residual tracer and formulation components, followed by air-drying in a fume hood to ensure complete evaporation. When coating was applied, the AINI was thoroughly cleaned with soap and water. Pipe cleaners were used to remove residual coating from inner surfaces, followed by rinsing with ethanol and air-drying.

### **Quantification of xylomethazoline and regional deposition of Otrivin**^®^

Xylomethazoline, the active pharmaceutical ingredient in Otrivin^®^, was quantified using a microDISS Profiler™ (Pion, MA, USA) equipped with 20 mm fiber-optic UV probes. Collected samples of Otrivin^®^ was diluted in PBS. Each sample was measured six times. A calibration curve was established using xylomethazoline standards in PBS ranging from 1 to 15 µg/mL. All measurements were performed at 230–240 nm using the second derivative.

The relative percentage distribution of xylomethazoline across nasal regions was calculated using the standard curve, assuming that the total recovered xylomethazoline represented 100% of the administered dose.

### **Dose volume estimation of Otrivin**^®^

The delivered dose volume of Otrivin^®^ was determined gravimetrically. Three individual Otrivin^®^ flasks were tested, with ten actuations per flask. Otrivin^®^ was sprayed into a pre-weighed Eppendorf tube, which was reweighed immediately after actuation. The dispensed volume per dose was calculated from the change in mass, assuming the formulation density was equivalent to that of water.

### Statistics

The regional distribution profiles were analyzed using JMP Pro 17 (JMP software, SAS institute, Copenhagen, Denmark) and GraphPad Prism 10.5.0 (774) (GraphPad Software, Boston, MA, USA). Interaction profiles and a prediction profiler were generated using the DoE analysis, including an effect summary with a 5% significance level.

## Results

The primary objective of the deposition analysis was to discover the optimal parameters which could maximize nasal spray delivery to the turbinate region of the AINI model and identify possible interactions between these parameters. The turbinate region was chosen, since it is the location of the respiratory mucosa which represents the most favorable site for drug uptake [3, 4].

### **Impact of airflow on Otrivin**^®^**distribution**

**Fig. 2:**
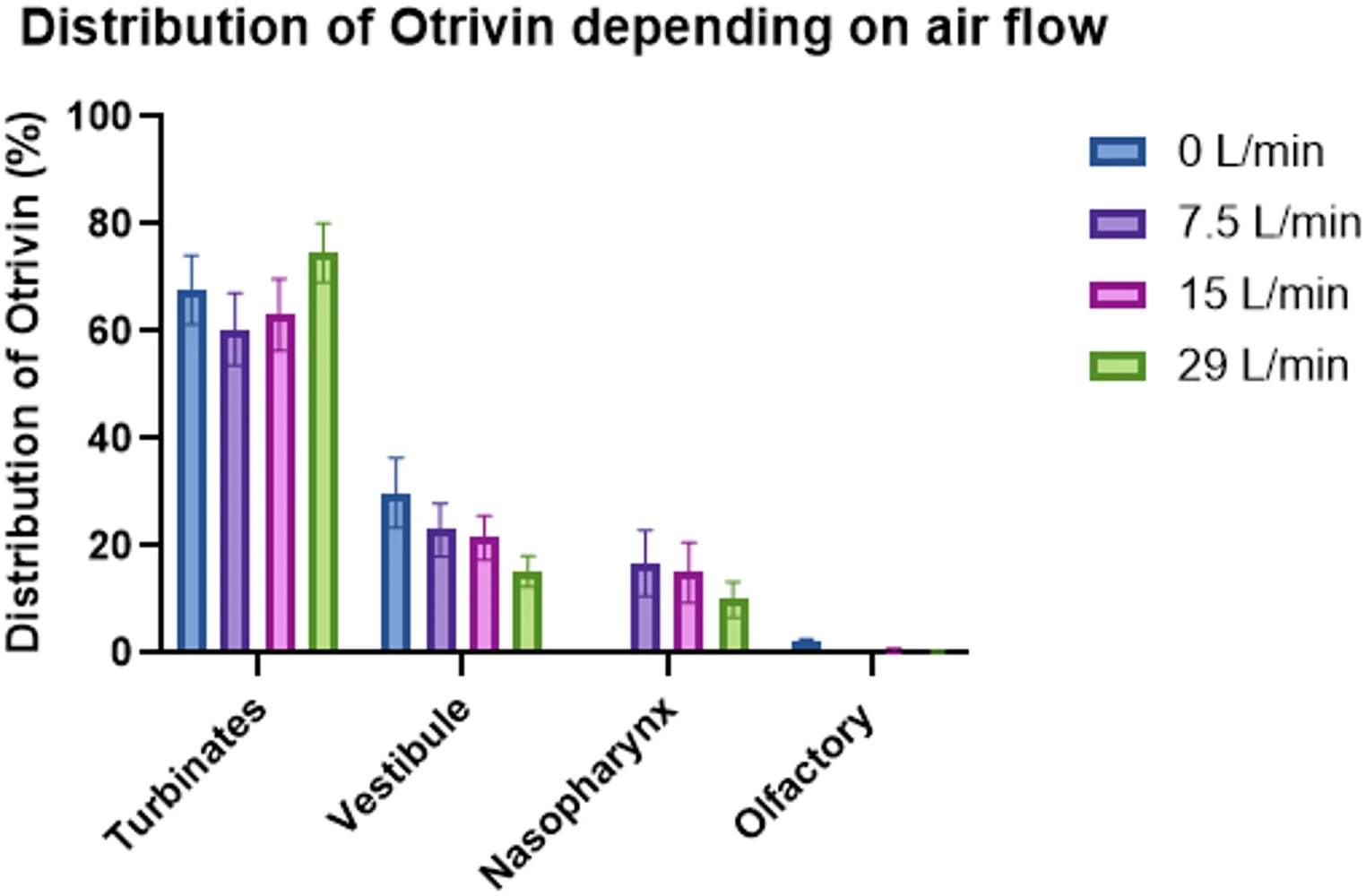
Distribution profile of Otrivin^®^ in the four regions: turbinates, vestibule, nasopharynx and olfactory, depending on the airflow. The mean ± SEM is plotted (*n* = 12)

The influence of airflow on Otrivin^®^ distribution was assessed by simulating varying respiratory conditions during administration. The results are shown in Fig. 2. An airflow of 0 L/min was used to represent breath-holding, while incremental flow rates up to 29 L/min simulated increasing levels of inhalation. Deposition in the turbinate region remained relatively stable across airflow conditions. An increase in nasopharyngeal deposition was observed when increasing the airflow from 0 L/min to 7.5 L/min, suggesting that the introduction of airflow facilitated particle transport deeper into the nasal cavity. At 29 L/min, a reduction in nasopharyngeal deposition was observed, compared to 7.5 and 15 L/min, indicating a shift in airflow dynamics. However, for turbinate deposition, no significant difference was observed between the different airflows (Fig. 5d).

### **Impact of actuation angle on Otrivin**^®^**distribution**

**Fig. 3:**
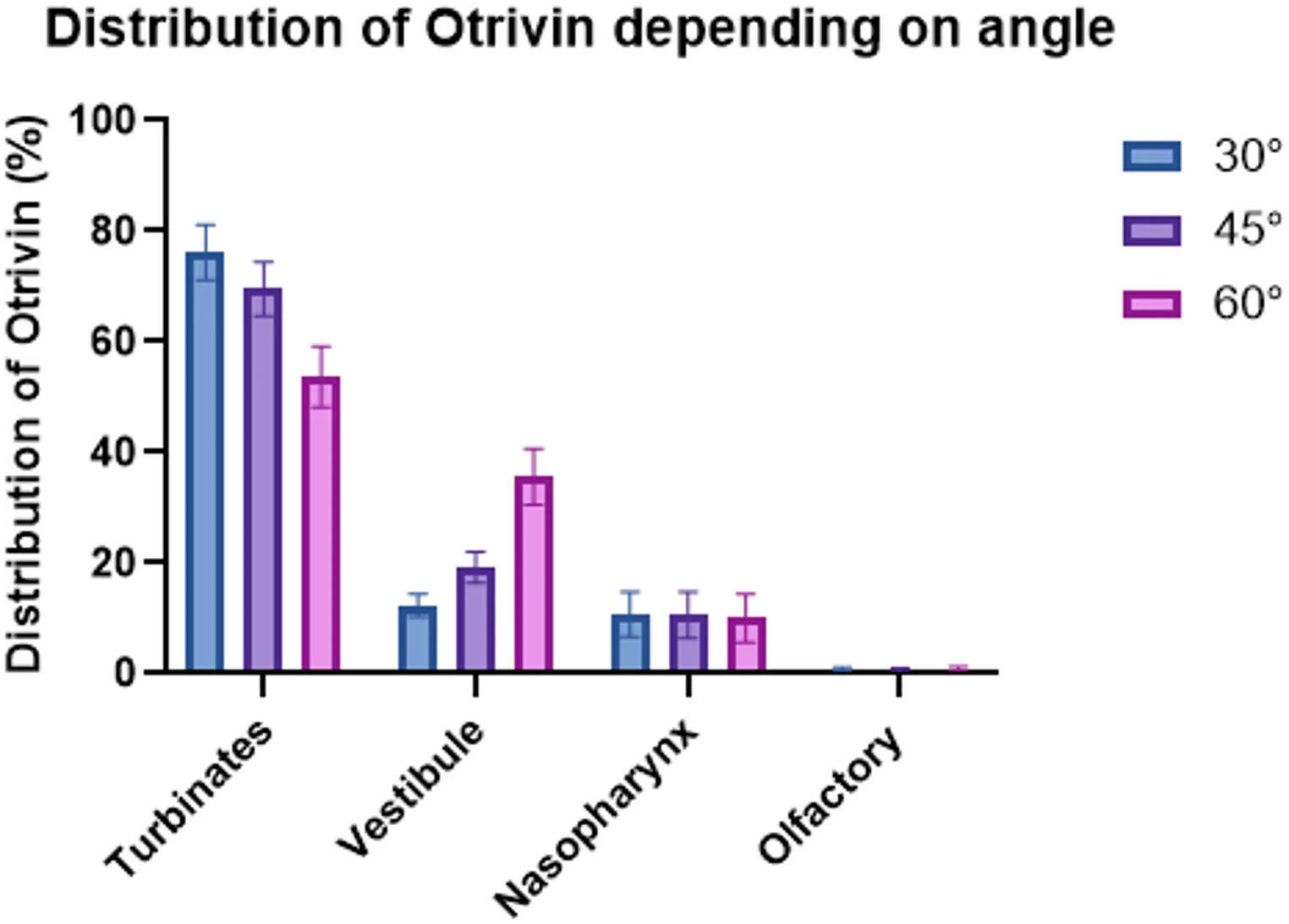
Distribution profile of Otrivin^®^ in the four regions: turbinates, vestibule, nasopharynx and olfactory, depending on the actuation angle. The mean ± SEM is plotted (*n* = 16)

The effect of actuation angle on Otrivin^®^ distribution was systematically evaluated (Fig. 3), given its known influence on deposition outcomes within the nasal cavity [9, 18]. Deposition in the olfactory and nasopharynx regions remained relatively constant across all angles tested. For the turbinate region, a pattern was observed, where increasing the actuation angle led to a decrease in turbinate deposition of Otrivin^®^. Conversely, for the vestibular region, an increase in the actuation angle led to an increased deposition of Otrivin^®^. An actuation angle of 30° was observed to result in the highest deposition in the turbinate region, along with the lowest deposition in the vestibule, compared to the two other angles. A significant difference in the turbinate deposition was observed between 30° and 60° (Fig. 5d).

### **Impact of temperature on Otrivin**^®^**distribution**

**Fig. 4:**
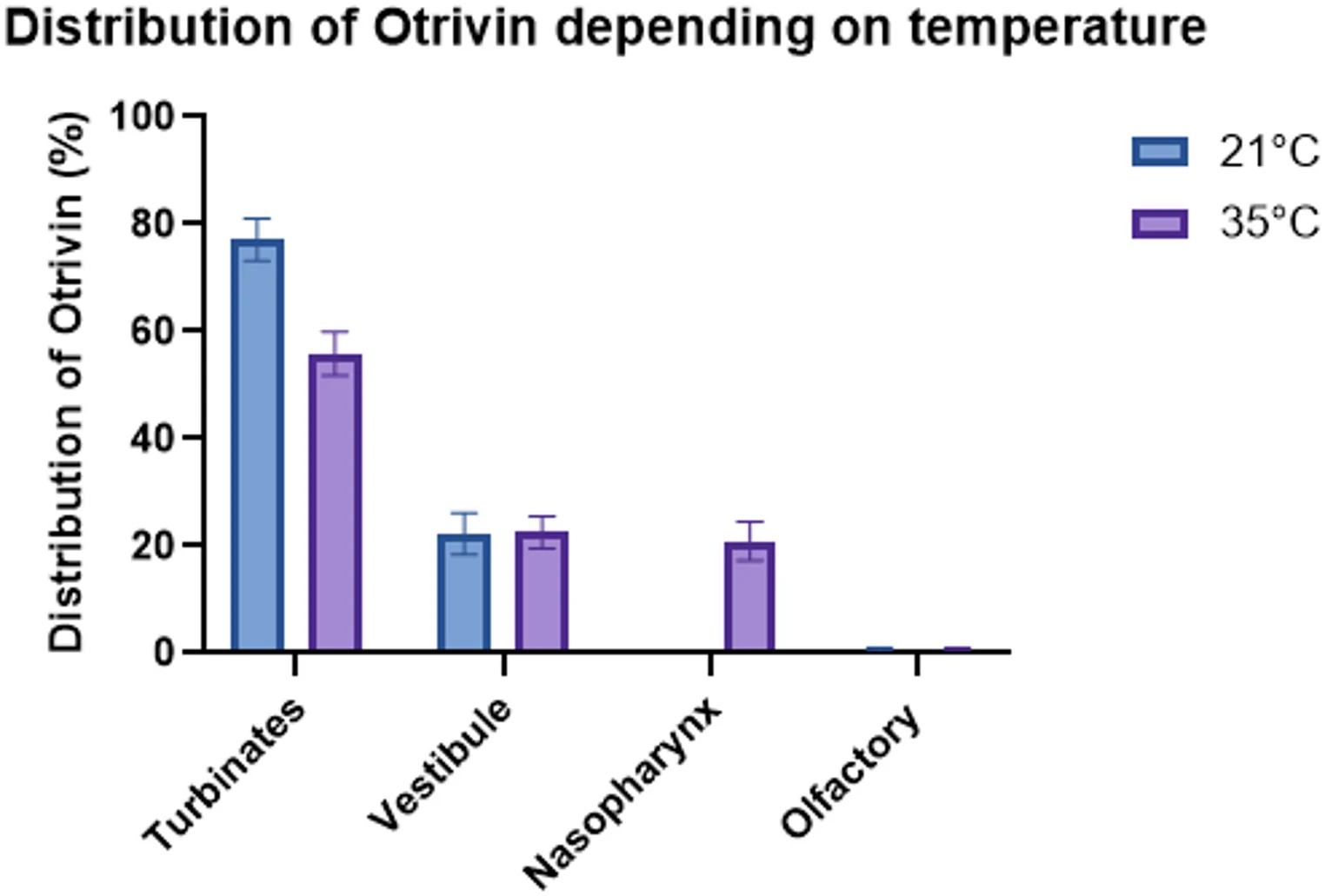
Distribution of Otrivin^®^ in the four regions: turbinates, vestibule, nasopharynx and olfactory, depending on the temperature. The mean ± SEM is plotted (*n* = 24)

**Fig. 5:**
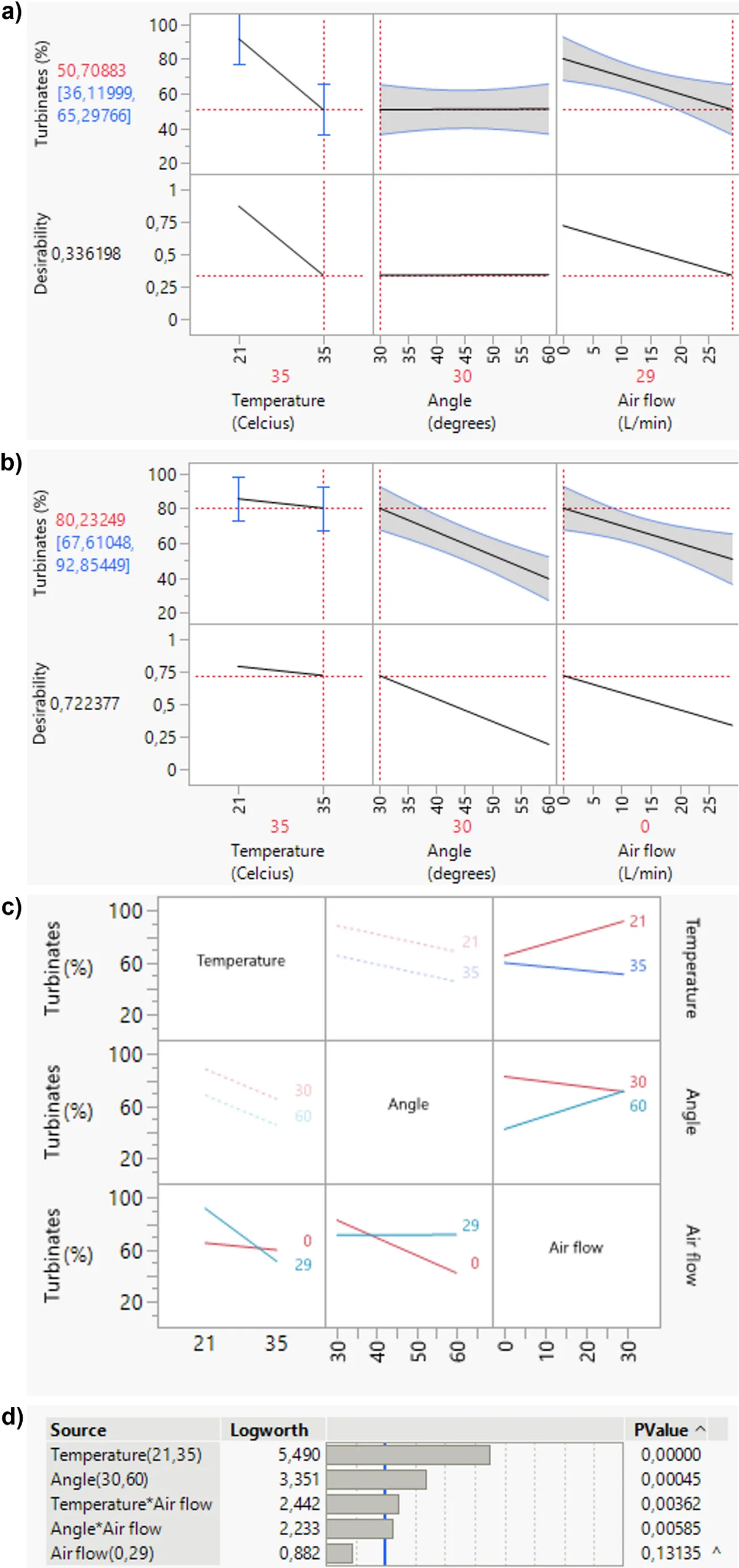
**(a)** Prediction profiler set at 35 °C, 30° and 29 L/min predicting an average turbinate deposition of 50.7% of Otrivin^®^, **(b)** prediction profiler set at 35 °C, 30° and 0 L/min predicting an average turbinate deposition of 80.2% turbinate deposition of Otrivin^®^, **(c)** interaction profiles between airflow, temperature and angle. Top right corner demonstrates significant interaction between temperature and airflow, the middle right corner demonstrates significant interaction between angle and airflow, the bottom left corner demonstrates significant interaction between temperature and airflow, and in the bottom row in the middle the significant interaction between airflow and angle is shown. No interaction was observed between temperature and angle, as demonstrated in top middle square and in the middle left corner with dotted parallel lines, **(d)** effect summary of the factors and two-factor interactions for deposition in the turbinates. A 5% significance level was used

The impact of temperature on the deposition profile in the AINI was investigated by conducting the experiments either at RT (21 °C) or at a physiologically relevant temperature (35 °C). As seen in Fig. 4, an increase in temperature was associated with a significant reduction in the amount of Otrivin^®^ (Fig. 5d) that deposited in the turbinate region. Furthermore, a corresponding increase in the nasopharynx was observed, indicating temperature-dependent changes in the spray dynamics where higher temperatures are associated with slightly lower spray viscosities and increased droplet velocity [19]. This resulted in a deeper deposition within the nasal cavity, whereas changes in the deposition pattern were observed for the turbinate and nasopharynx region. The deposition in the vestibule and olfactory regions remained relatively constant regardless of the temperature, with minimal deposition in the olfactory region.

### **Influence of combined parameters on Otrivin**^®^**distribution in the turbinates**

For the influence of combined parameters, the obtained data showed a significant interaction between temperature and airflow, as well as between airflow and angle (Fig. 5c-d). From the prediction profiler, it was possible to assess the predicted deposition in the turbinates at all the tested parameters. Since 35 °C was identified as the most relevant test temperature to simulate in vivo conditions, and the 30° actuation angle resulted in the highest deposition in the turbinates (Fig. 3), along with a significant interaction between airflow and angle, two possible outcomes at 35 °C and 30° under different airflow conditions are presented in Fig. 5a-b. The DoE analysis showed that the predictive model was statistically valid and fitted the data sufficiently with no significant lack of fit (*p* = 0.163) and the residuals were randomly distributed around zero without noticable patterns. While the model was significant (*p* < 0.0001), its predictive power was moderate (R^2^ = 0.6 and maximum achievable R^2^ = 0.81), meaning that it captured the main trends, however with some variability (RMSE ≈ 14.8%). There were no strong outliers, suggesting that the model form was appropriate. The limitations in prediction were likely due to experimental noise and unmeasured factors rather than problems with the model itself, and the model still explained 74% (dividing model R^2^ with maximum achievable R^2^) of the maximum explainable variation.

The prediction profiler predicted an average deposition in the turbinates of 50.7% at 29 L/min, whereas it predicted an average deposition in the turbinates of 80.2% at 0 L/min, a relationship in accordance with the interaction profiler (Fig. 5c).

### Effects of Brij coating

Following the DoE analysis, the effect of Brij coating was investigated at an angle of 30° and an airflow of 29 L/min, at 21 °C and 35 °C, respectively. The angle of 30° was chosen since it showed the highest deposition in the turbinate region, compared to the two other angles (Fig. 3). Although, the interaction profiler (Fig. 5c) showed a higher deposition in the turbinates at 0 L/min than at 29 L/min (at 30°), 29 L/min was chosen to simulate breathing effects and since this was a recommended flow rate from the supplier of the AINI (Copley Scientific).

**Fig. 6:**
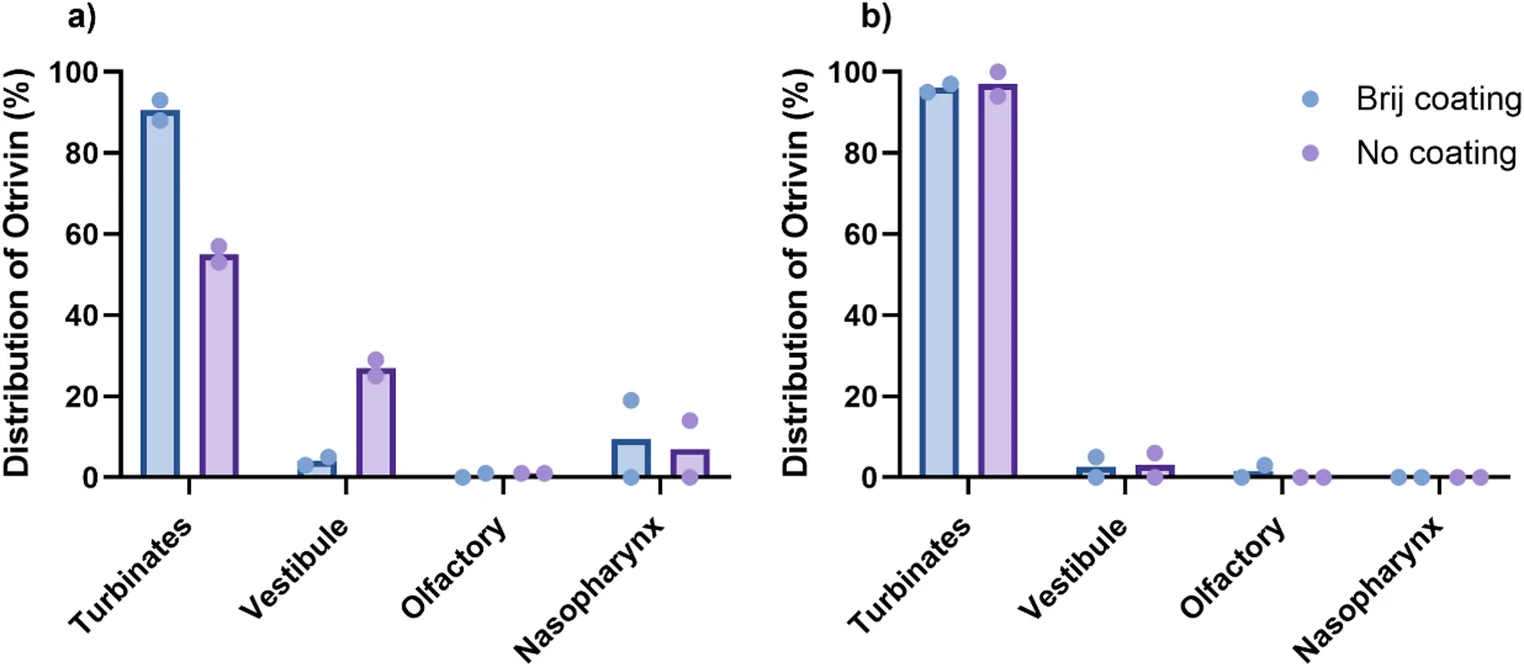
**(a)** Coating vs. no coating at 35 °C, 30° and 29 L/min, **(b)** coating vs. no coating at 21 °C, 30° and 29 L/min. The mean is plotted (*n* = 2)

In Fig. 6, the effect of applying a coating in the AINI is highlighted at 21 °C and 35 °C. An increased deposition in the turbinate region was observed when coating was applied at 35 °C, along with a reduced deposition in the vestibular region. The difference in deposition pattern depending on the coating was not observed at 21 °C, where turbinate deposition remained relatively high for both experiments with and without coating.

### Recovery from AINI

A concentration of 1 mg/mL of xylomethazoline in Otrivin^®^, as well as a volume of one spray of 140 µL, was acquired from the Otrivin^®^ package information. Therefore, a 100% recovery was presumed to be 140 µg, assuming that the formulation density was equivalent to that of water. The recovered Otrivin^®^ from all the experiments were 104.9 ± 17.9 µg. To determine the recovery of Otrivin^®^ from the AINI, sprays masses from three different Otrivin^®^ sprays were measured (*n =* 10). The values are outlined in Table 3.

**Table 3:** Overview of Otrivin^®^ dose mass (*n* = 10). Mean ± SD is highlighted

Otrivin 1	Otrivin 2	Otrivin 3
134 ± 2.8	143 ± 5.5	130 ± 12.8

Taking the average of the spray masses from the three different Otrivin^®^ sprays and comparing with the average of the recovered Otrivin^®^ from the experiments, the recovery from the AINI was calculated to 77.3%.

## Discussion

Various nasal cast models have been investigated to assess the regional deposition of nasal spray formulations [9–11]. This study aimed to investigate how selected parameters influenced the nasal spray (Otrivin^®^) deposition within the AINI, with a focus on deposition in the turbinate region.

Using a DoE approach, we investigated how different parameters influenced nasal spray deposition in the AINI model. We aimed to uncover the main effects of the individual parameters, as well as potential interactions between these parameters. Additionally, we tested the impact of applying a mucus-simulating coating in the AINI at 30°, 29 L/min, at both 21 °C and 35 °C.

We found that temperature and angle of administration (30° and 60°) significantly affected turbinate deposition, while airflow alone did not have a significant effect. However, interactions between airflow and temperature, as well as airflow and angle, were significant. Additionally, we observed that coating the AINI at 35 °C increased turbinate deposition compared to uncoated conditions, and an overall Otrivin^®^ recovery of 77.3% was achieved. The remaining 22.7% unable to be collected and quantified could be due to dripping of the nasal spray from the vestibule and limitations concerning the lower limit of detection by the quantification method, as well as the collection method.

The results of this study suggest that temperature is a critical factor for in vitro testing of nasal spray formulations. To our knowledge, this study is the first to incorporate physiologically relevant temperatures for regional deposition testing in vitro*.*

Higher temperatures increase molecular kinetic energy and reduce formulation viscosity [19], thereby altering droplet energy and enhancing transport into deeper regions. This was observed with a reduced deposition in the turbinates and an increased deposition in the nasopharynx at 35 °C, compared to 21 °C (Fig. 4). This mechanistic explanation supports the statistical finding that temperature is an important factor for the deposition pattern. Although, no significant effect of airflow alone was observed, as also previously found in literature [20–22], an interaction between temperature and airflow was observed. The interaction between temperature and airflow indicated that at 21 °C, the effect of airflow was more pronounced (Fig. 5c top right corner), as increasing the airflow enhanced turbinate deposition. At an airflow of 0 L/min, the turbinate deposition was comparable between 21 °C and 35 °C, whereas at the highest airflow (29 L/min), increasing temperature (21 °C to 35 °C) reduced the deposition in the turbinate region (Fig. 5c bottom left corner). These results suggest that the influence of airflow depends on the thermal state of the formulation.

The main effect of temperature and the combined interaction with airflow indicate the need to consider thermal conditions when conducting deposition studies in vitro.

For the angle of administration, deposition in the olfactory and nasopharyngeal regions remained constant across all angles (Fig. 5d), suggesting limited sensitivity of these areas to directional variation. This supports the previous findings of Suman et al., who observed that nasopharyngeal penetration is minimally influenced by plume orientation [18]. The inverse relationship between actuation angle and turbinate deposition could be explained by alterations in spray trajectory. At 30°, the spray plume is directed towards the deeper nasal cavity, facilitating direct turbinate access. In contrast, at 60°, the plume is directed upward, thereby increasing interaction with anterior surfaces, such as the vestibule, before reaching deeper regions. A similar relationship was observed by Costa et al. [23]. In general, it has been found that a lower administration angle (< 45°), where the spray nozzle is aimed directly at the nasal valve, maximizes deposition in the turbinates [11, 20, 21]. Although, previous studies have emphasized the importance of the actuation angle [9, 18], differences in geometric definitions between models limit direct comparison, such as methodological differences in model structures and how regional compartments are defined across nasal cavity simulations. However, previously it has been found that deposition of xylomethazoline nasal spray in a nasal cast could be altered with the angle of administration, where a 30° angle resulted in higher deposition in the turbinates, whereas a 60° angle led to increased deposition in the frontal cavity of the nasal cast. The effect of breathing compared to no breathing simulation was also found to alter the deposition area, where breathing effects significantly reduced deposition area, possibly due to an increased airflow leading to turbulence and dragging of the droplets in unpredictable directions leading to a decreased nasal deposition [24].

At 30° administration, increasing the airflow slightly reduced turbinate deposition, suggesting that airflow redirects the plume away from its initial deep trajectory. However, at 60°, airflow aided in overcoming the anatomical curvature, thereby increasing turbinate deposition (Fig. 5c middle right corner). This reinforces the notion that airflow only modulates plume transport when combined with geometric orientation. At an airflow of 29 L/min, the effect of angle was negligible, indicating that the aerodynamic forces are strong enough to carry the spray toward the turbinate region irrespective of the initial trajectory. Conversely, at an airflow of 0 L/min, increasing the actuation angle resulted in a decline in the turbinate deposition (Fig. 5c bottom row in the middle), suggesting that without the assistance of airflow, steeper angles may redirect the spray toward anterior or superior regions of the nasal cavity, thereby reducing deposition in the turbinates.

Coating the internal regions of the AINI only affected the deposition pattern at 35 °C, resulting in an increased deposition in the turbinates, which was likely due to reduced droplet rebouncing and enhanced surface adhesion, which has previously been found to contribute to localized deposition at the initial contact sites [15, 16]. At 21 °C, coating did not alter deposition patterns as turbinate deposition was already high without coating. Brij 35 has a melting point at 43 °C and is therefore considered stable at both temperatures [25]. This temperature dependence further confirms that the thermal state plays a central role. At physiological temperature, without coating, nasal spray deposition in the turbinates decreased with increasing flow rates (Fig. 5c). With the addition of coating, this effect was minimized, leading to a higher deposition in the turbinates, probably due to reduced droplet rebouncing and enhanced surface adhesion.

However, some limitations are worth noting. The coating process of the AINI was achieved using pipe cleaners immersed in the coating solution. More controlled coating strategies should be used in future work to obtain a direct quantification of the amount of coating solution applied, as well as quantification of the thickness of the coating and how this correlate to in vivo conditions.

The need for assessing combined interactions between parameters is highlighted in this study. As discussed by Chen et al. [5], interactions between different parameters might strongly affect deposition patterns and to a stronger degree than if these parameters are considered in isolation. Therefore, we believe it is critical to assess these parameters in connection to each other in order to understand deposition patterns, and to understand the interactions between these parameters. However, subject variability aspects of deposition patterns should be considered as a limitation when only one nasal cast is used to determine deposition characteristics, since deposition patterns have previously been observed to differ significantly within different subjects [26]. Another important aspect to keep in mind when conducting deposition studies of nasal spray formulations in vitro is the aspect of mucociliary clearance. It is worth considering that mucociliary clearance can affect the subsequent drug uptake, after deposition in a certain area, which has been found to be related to particle size and whether deposition is in ciliated or unciliated areas [27, 28].

Although the results from this study highlight the influence of three main parameters, more research is needed for investigating regional deposition in vitro that also includes other factors such as spray pattern and particle size distribution.

## Conclusion

In this study, the influence of administration angle, airflow, and temperature on nasal spray deposition was investigated in the AINI using a DoE approach. Especially the use of a physiologically relevant temperature condition noticeably altered deposition patterns, highlighting the critical role of the thermal state in governing spray transport and regional targeting. The findings from this study emphasize that in vitro nasal deposition studies conducted at room temperature may not accurately reflect in vivo behavior. Furthermore, the observed interactions between airflow and temperature, and airflow and angle underscore the significant importance of incorporating multifactorial experimental designs when evaluating nasal drug delivery performance.

## Supplementary Information

Below is the link to the electronic supplementary material.

## Data Availability

The data generated in this study is available from the corresponding author upon reasonable request.
