# Supplementary material for: A design of experiments study of nasal spray deposition in the alberta idealized nasal inlet
Source: Drug Deliv Transl Res. 2026 May 6;16(9):3015–25. doi: 10.1007/s13346-026-02147-0 (PMC13538200; doi:10.1007/s13346-026-02147-0)
Supplement: Supplementary file 1 [file 13346_2026_2147_MOESM1_ESM.docx]

**Supplementary information**

**Figure S1**: Figure showing how the, **a+b)** vestibule, **c+d)** turbinates, **e)** olfactory region, and **f+g)** nasopharynx were covered with parafilm and tape for sample collection.
